# Maternofetal outcomes of obstructed labor among women who gave birth at general hospital in Ethiopia

**DOI:** 10.1186/s13104-019-4165-8

**Published:** 2019-03-12

**Authors:** Tewodros Eshete Wonde, Abebe Mihretie

**Affiliations:** 1 Department of Public Health, College of Medicine and Health Sciences (CMHS), Debre Markos University (DMU), Debre Markos, Ethiopia; 2 Department of Midwifery, College of Medicine and Health Sciences (CMHS), Debre Brhan University, Debrebrhan, Ethiopia

**Keywords:** Obstructed labor, Mal-presentation, Cephalo pelvic disproportion

## Abstract

**Objective:**

Obstructed labor had different maternal outcomes such as uterine rupture, postpartum hemorrhage, puerperal sepsis, Vesico-Vaginal fistula (VVF), recto-vaginal fistula can leads to death. Besides fetal outcomes including birth asphyxia, still birth, neonatal jaundice and umbilical sepsis can occur. Identifying maternal and fetal outcomes of obstructed labor among women who gave birth at Suhul general Hospital, Shirie town, Tigray, Ethiopia has been done using a retrospective review of delivery charts and registration book.

**Results:**

Majority of mothers 69 (75.8%) came from rural areas and 74.7% were married. Cephalo pelvic disproportion occurs in 59 (64.8%) and mal-presentation in 28 (30.8%) of obstructed labor. Fetal congenital anomaly (hydrocephalus) occurs in 3 (3.3%) of cases and pelvic mass constituted 1 (1.1%) of cause of obstructed labor. Above half of mothers delivered with obstructed labor had sepsis 23 (25.3%), post-partum hemorrhage 10 (11%), Vesico Vaginal Fistula 5 (5.5%) and anemia 15 (16.5%). From the well-known causes of obstructed labor; mal-presentation, Cephalo pelvic disproportion, fetal congenital anomaly, and pelvic mass were found to the common outcomes of obstructed labor in our study area. Besides Still birth, birth asphyxia, and birth injury were the others.

## Introduction

Obstructed labor is defined as failure of the fetal presenting part to descent in the birth canal due to mechanical reasons, adequate, and strong uterine contraction it leads to various maternal and fetal outcomes [1–3].

In trends of maternal mortality from 1990 to 2013, there is the reduction of maternal mortality ratio (MMR) by 45% in all Millennium Development Goal (MDG) regions of the world. But there is still high amount of MMR, which is 210 per 100,000 live births or 289,000 maternal deaths in 183 countries globally. From this, developing countries account for 99% maternal deaths and 62% maternal deaths occurred in Sub Saharan region [4].

Even though, Ethiopia reduced MMR to 676 per 100,000 live births in 2010 from 871 per 100,000 live births in 2000, still it had the highest maternal mortality ratio in Africa. And the country is clearly off the track in goal 5 because of unable to reduce MMR to 267 per 100,000 live births in 2015 based on its plan. Delays in seeking skilled emergency obstetric care; delays in reaching the health facility, delays in receiving a timely intervention after reaching the facility, low skilled birth attendant and large proportions of unmet family planning needs among girls in child-bearing ages are mentioned as major factors for this unsuccessful achievement towards MDG5. Obstructed or prolonged labor, postpartum hemorrhage, infections, ruptured uterus, severe preeclampsia and unsafe abortion were the major direct causes of maternal death and from these causes, except sever preeclampsia and unsafe abortion the others were direct complications of obstructed labor [5–7].

Obstructed labor has different magnitude in different developing countries ranging from 2 to 8%. When we come to Africa some research finding showed that the magnitude of obstructed labor was more than the above determined once; In Uganda and Ethiopia the magnitude of obstructed labor was described as 10.5% and 12.2% respectively [3, 8].

About 80% of global maternal deaths were contributed by pregnancy induced hyper tension and Obstructed labor with its direct complications such as hemorrhage, sepsis and uterine rupture [5]. According to WHO world health report of 2005, obstructed labor accounts about 8% of maternal deaths globally, and its morbidity and mortality impact was increases in developing countries, as evidenced by some studies, it causes about 3.5% and 14% maternal deaths in Ethiopia and Nigeria respectively [1, 2, 9].

Apart from maternal deaths, obstructed labor had different maternal outcomes such as uterine rupture, postpartum hemorrhage, puerperal sepsis, Vesico-Vaginal fistula (VVF), recto-vaginal fistula (RVF) and fetal outcomes including birth asphyxia, still birth, neonatal jaundice and umbilical sepsis [10–13]. Women who experienced obstructed labor for prolonged time can be complicated with fistula. Besides of their physical wounds, serious social issues of divorce, separation from religious exercises, detachment from their families which can worsen poverty and malnutrition, are the major problems of obstructed labor [13].

## Main text

### Methods

#### Participants and setting

The study was conducted in Suhul General Hospital in 2015. Maternal charts for those mothers who gave birth at Suhul general hospital from September 2012 to August 2015 were studied. There were 91 obstructed labor maternal charts that had the diagnosis of obstructed labor for those mothers who gave birth at suhul general hospital. We have included maternal charts, for those mothers, who gave birth in normal vaginal and operative deliveries during the study period satisfied the inclusion criteria. Besides, maternal charts for those mothers who gave birth with elective c/s and charts without medical record number were excluded from the study.

#### Data collection methods

Secondary data was collected primarily by reviewing parturient chart with prepared checklist. If adequate data was not obtained from the parturient chart, delivery registration book and operation registration book were used to fulfill the remaining information.

The data quality was controlled by doing pretest in about 10% of the sample and providing training for every data collectors before the actual data collection was conducted. Close supervision of the data collectors during the data collection period was another means of data quality control and each checklist was checked for its completeness daily. Data collectors were four Degree midwife staffs who work at Suhul general hospital delivery unit in different shifts.

### Results

#### Socio demographic characteristics of the women

A total of 91 maternal charts with obstructed labor were studied. The mean age and standard deviation were 25.4 ± 7.7, while majority of mothers (25.3%) were found in the age range of 25–29 years old. With regard to maternal marital status majority of mothers 68 (74.7%) were married, but about 19 (20.9%) had no registration that can describe whether they are married or not and they were described as unknown marital status (Table 1).

**Table 1:** Socio demographic characteristics of the women at Suhul general hospital, in north west zone, Shirie town, Tigray, Ethiopia, from September 2012 to August 2015

Variable	Obstructed labour (n = 91)
*Age in years*	
< 20	17 (18.9%)
20–24	15 (16.5%)
25–29	23 (25.3%)
30–34	21 (23.1%)
35–39	9 (9.9%)
≥ 40	6 (6.6%)
*Residence*	
Rural	69 (75.8%)
Urban	22 (24.2%)
*Referral site*	
Direct coming	3 (3.3%)
Referred from HC	85 (93.4%)
Referred from hospital	3 (3.3%)
*Marital status*	
Married	68 (74.7%)
Single	4 (4.4%)
Unknown	19 (20.9%)

#### Obstetrical characteristics of the women

Majority of the mothers were primiparas, which was 40 (44%) whereas, grand multipara mothers were accounts for 19 (20.9%). With regard to ANC follow up about three-fourth of mothers had three or less ANC follow up, which was 67 (73.6%). Only 24 (26.4%) of mothers had regular ANC visit among cases. In related to gestational age 43 (47.3%) of the women were found in term gestational age, (37 weeks–40 weeks). The mean duration of labor among mothers was 26.9 ± 15.2 h with the range of 11–72 h. About 13 (14.3%) of mothers had ever different obstetrical problems such as, anti-partum hemorrhage, premature rupture of membrane, pre term labor, twin pregnancy, hypertension problems and others (Table 2).

**Table 2:** Obstetrical characteristics of the women at Suhul general hospital, in north west zone, Shirie town, Tigray, Ethiopia, from September 2012 to August, 2015

Variable	Labour obstructed (n = 91)
*Parity*	
Para 1	40 (44%)
Para 2–4	32 (35.2%)
Para ≥ 5	19 (20.8%)
*History of still birth*	
Yes	13 (14.3%)
No	78 (85.7%)
*History of c/s*	
Yes	5 (5.5%)
No	86 (94.5%)
*ANC follow up*	
≤ 3 visit	67 (73.6%)
≥4 visit	24 (26.4%)
*Gestational age*	
< 37 weeks	5 (5.5%)
37–40 weeks	43 (47.3%)
41–42 weeks	23 (25.3%)
> 42 weeks	8 (8.8%)
Unknown GA	12 (11%)
*Labor duration (h)*	
< 12	1 (1.1%)
12–18	39 (42.9%)
19–24	26 (28.6%)
> 24	25 (27.5%)
*Any obstetrical problem*	
Yes	13 (14.3%)
No	78 (85.7%)
*Obstetrical problems*	
APH	2 (2.2%)
PROM	3 (3.3%)
Pre term labor	2 (2.2%)
Twin pregnancy	3 (3.3%)
Hyper tension	2 (2.2%)
Other^a^	1 (1.1%)
No problem	78 (85.7%)
*Fetal presentation*	
Vertex	63 (69.2%)
Breach	9 (9.9%)
Shoulder	7 (7.7%)
Face	5 (5.5%)
Other	7 (7.7%)

^a^Prolapsed cord, polyhydramnios and intra uterine fetal death

#### Causes of obstructed labor

Cephalo pelvic disproportion (CPD), mal-presentation, congenital anomaly and pelvic mass were assessed as the causes of obstructed labor in this study. From these CPD and mal-presentations were the commonest one. CPD occurs in 59 (64.8%) and mal-presentation occurs in 28 (30.8%) cases. Fetal congenital anomaly (hydrocephalus) occurs in 3 (3.3%) of cases and pelvic mass constituted 1 (1.1%) of cause of obstructed labor.

#### Perinatal outcomes

Following obstructed labor there were many perinatal outcomes and from this study around 49 (52.1%) neonates had birth asphyxia with APGAR score of less than four at 5th minutes after birth, 34 (36.2%) were born as still birth and 1 (1.1%) neonate had birth injury (cranial injury) during operative delivery. Only 10 (10.6%) of neonates had an APGAR score of ≥ 7 at 5th minutes after birth from these obstructed laboring mothers.

#### Maternal outcomes

Different maternal outcomes were observed among obstructed laboring mothers. From these, about 8 (8.8%), 15 (16.5%) and 10 (11%) of them had bladder trauma, ruptured uterus and hysterectomy respectively. Additionally sepsis, post-partum hemorrhage, VVF and anemia were developed in 23 (25.3%), 10 (11%), 5 (5.5%) and 15 (16.5%) mothers respectively. Around 20 (22%) of women from obstructed labor did not develop any complication.

### Discussion

In this study, majority of the cases 75.8% were rural resident, nearly similar with the study result of Hawassa in which about 65.3% of the cases were from rural areas [11]. Additionally, descriptive studies in Adigrat, Pakistan and Bangladesh also mentioned that majority of mothers who had obstructed labor were from rural resident, which was similar to this study finding [1, 13, 14].

In this study about three-fourth of the mothers (73.6%) had irregular ANC follow up, almost similar with the study conducted in west Wollega, that revealed around half of the mothers (48.3%) had irregular visit and around 12% of them had unknown frequency of visit [10]. But this result was contradicted with the study results of Adigrat, Nigeria, Jimma, Pakistan, and Zaire in which most of the cases had no ANC visit [1, 2, 8, 13, 14]. This difference might be due to time variation, in which current ANC coverage was increased in Ethiopia than before.

In this study majority of the cases (44%) were primiparas. This result was concurred with the study result of Hawassa, Pakistan, Bangladesh and Uganda in which most of the cases were primiparas [3, 11, 13, 14]. Around 14.3% of mothers with obstructed labor had passed thorough different obstetrical problems such as Ant Partum Hemorrhage, Pre mature Rupture of Membrane (PROM), hypertension and Twin pregnancy. This is nearly similar with the studies done in west Uganda and Hawassa, in which about 4.4% of study participants had twin pregnancy and 2.8% of them had PROM respectively [3, 11].

Obstructed labor with different maternal outcome was assessed; from this sepsis, uterine rupture and anemia were the commonest in about 25.3%, 16.5% and 17% of the cases respectively. This study was almost similar with the study results of Adigrat, Jimma and Zaire in which most of the cases develop at least one complication [1, 8, 14].

With regard to perinatal outcome, about 32.6% of the newborns had still birth. It is relatively low when compared with the study results of Adigrat and Jimma, in which about 50.3% and 54.2% of the newborns had still birth respectively [1, 8]. This might be due to the fact that variation in duration of labor. The mean duration of labor was 26.9 ± 15.7 h with the maximum of 72 h for this study and 45.4 ± 21.7 h with the maximum of 144 h for Adigrat and about 40.8% of the cases had labor duration of > 24 h in the study of Jimma [1, 8]. Hence, as the duration of labor was increased, the burden of complication would be increased.

### Conclusion

In this study we have identified different maternal and perinatal outcomes of obstructed labor.

From the well-known causes of obstructed labor; Mal-presentation, Cephalo pelvic disproportion, fetal congenital anomaly and pelvic mass were found to the common outcomes of obstructed labor in our study area. Besides Still birth, birth asphyxia and birth injury were another perinatal outcomes following obstructed labor.

Furthermore, different outcomes such as uterine rupture, maternal sepsis, anemia, post-partum hemorrhage, bladder injury and fistula were assessed from mothers chart as the maternal problems that come up following obstructed labor.

We recommend the Health institution to give immediate feedback for those health institutions that refer obstructed laboring mothers and supervise them in outreach programme. Health professionals have better to give emphasis about obstructed labor complications for each pregnant mother during ANC visits. Furthermore, health extension workers have to adequately work on teaching the community about the adverse maternal and fetal outcome of obstructed labor.

## Limitation of the study

Because of using secondary data some variables were missed.

## Abbreviations

ANC: anti natal care
APH: ant partum hemorrhage
CPD: cephalo pelvic disproportion
HC: Health Center
MDG: Millennium Development Goal
MMR: maternal mortality ratio
PROM: pre mature rupture of membrane
RVF: recto vaginal fistula
VVF: vesico vaginal fistula
WHO: World Health Organization
